# Reframing physical literacy for lifelong movement and societal wellbeing: a habit-informed perspective

**DOI:** 10.3389/fspor.2026.1849551

**Published:** 2026-07-24

**Authors:** Nigel Green, Kyle Pushkarenko

**Affiliations:** 1 IPLA, Wigan, United Kingdom; 2 Memorial University of Newfoundland, St. John’s, NL, Canada

**Keywords:** habits, lifelong movement, physical activity, physical literacy, wellbeing

## Abstract

Physical inactivity remains a persistent global challenge with implications for physical, cognitive, emotional, and social wellbeing across the lifecourse. Physical literacy has become an influential framework for understanding lifelong engagement in movement, yet less attention has been given to how such engagement becomes sustained, interrupted, and renewed in everyday life. This conceptual paper develops a habit-informed perspective on physical literacy by examining the recurring patterns through which people come to notice, value, prioritise, and return to movement across contexts and life stages. Drawing on habit theory, identity-based motivation, and physical literacy scholarship, the paper argues that sustained engagement in movement depends not only on capability, knowledge, or motivation, but also on repeated ways of thinking, feeling, relating, and acting. Stephen Covey's Seven Habits are used as a heuristic scaffold for naming pedagogically relevant patterns, including agency, purpose, prioritisation, inclusive participation, empathy, collaboration, and renewal. The paper further situates physical literacy as relevant to the Inner Development Goals and selected United Nations Sustainable Development Goals by showing how physical education may support inner capacities and broader societal aims related to health, education, equity, and sustainable communities. Physical literacy is therefore reframed as a context-sensitive relationship with movement that is shaped by biography, social relationships, access, opportunity, and repeated educational experiences. Implications are outlined for physical education pedagogy, curriculum, and research, with emphasis on supporting lifelong movement in ways that are meaningful, inclusive, and socially responsive.

## Introduction—physical literacy, inactivity, and the need for a habit-informed perspective

Physical inactivity remains a major global health concern. Large proportions of children, adolescents, and adults do not meet recommended physical activity levels, with implications for noncommunicable disease, mental health, social participation, and overall wellbeing across the lifecourse (1–3). These concerns are also reflected in broader global agendas, including the United Nations Sustainable Development Goals, which foreground health and wellbeing, quality education, gender equality, reduced inequalities, and sustainable communities as interconnected societal priorities (4). Physical inactivity is therefore not only an individual behavioural issue; it is also shaped by social, educational, environmental, and policy conditions that influence whether people have meaningful opportunities to participate in movement (5). In this context, physical literacy offers a useful framework for understanding how people develop and sustain meaningful relationships with movement. Whitehead defines physical literacy as “the motivation, confidence, physical competence, knowledge and understanding to value and take responsibility for engagement in physical activities for life” (6). This definition positions physical literacy as more than physical activity volume or motor performance alone. It foregrounds how people come to value, interpret, and take responsibility for movement across changing life circumstances. Although the philosophical roots of physical literacy remain important, the present paper focuses less on re-establishing those foundations and more on clarifying how physical education may support the patterns through which physical literacy is sustained across the lifecourse.

Viewed in this way, physical literacy is best understood as relational and ongoing rather than as a fixed possession or end-state (7, 8). Its development unfolds across the lifecourse through what might be termed a movement biography: the accumulated experiences, opportunities, relationships, expectations, and constraints that sediment over time and shape future engagement (9, 10). Yet despite physical literacy's emphasis on lifelong engagement, an important question remains underdeveloped: how does a relationship with movement become sufficiently patterned, resilient, and personally meaningful to endure across changing contexts, transitions, interruptions, and life stages (8, 11)? Much of the literature has focused on domains, philosophical foundations, and assessment, while comparatively less attention has been given to the patterns of thinking, feeling, relating and acting that help people continue to choose, prioritise, and return to movement in everyday life.

Here, a habit-informed perspective offers a useful conceptual entry point. Drawing on Dewey (12), Lally et al. (13), and Wood and Rünger (14), habits can be understood as patterned ways of thinking and acting that become stabilised through repetition and context. In this paper, however, habit is not treated as mindless routine or as a purely behaviourist mechanism. Rather, it is used to name the recurring, embodied, and socially shaped patterns of thinking, feeling, relating, and acting through which people come to approach movement, for example by noticing movement opportunities, protecting time for activity, seeking meaningful challenge, or responding to disruption with adaptation rather than withdrawal. Framing physical literacy in these terms helps shift the discussion from what physical literacy includes to how it is lived and sustained, opening a different set of questions about how movement-related orientations are formed, maintained, and pedagogically supported across the lifecourse. Throughout the paper, therefore, habit is used to illuminate processes of stabilisation, while recurring patterns of thinking, feeling, relating and acting describe how physical literacy is lived, sustained, and renewed across time.

### Purpose and questions

This paper argues that physical literacy is not only about what individuals know or can do, but also about how they characteristically come to think, feel, move and connect in relation to movement. Behaviour is shaped by habitual processes and automaticity (15, 16), but also by identity-based motivations that influence what actions feel congruent with the self (17). A habit-informed lens therefore offers a way of examining how movement becomes part of everyday life without collapsing physical literacy into simple compliance or repetition.

The purpose of this paper, therefore, is to develop a habit-informed perspective on physical literacy by drawing on Covey's (18) Seven Habits as a heuristic framework and situating that framework alongside the Inner Development Goals [IDGs; (19)] and the United Nations Sustainable Development Goals [SDGs; (4)]. Although the broader rationale for the paper is connected to physical inactivity and lifelong movement, the primary pedagogical focus is physical education. This focus is intentional. Physical education provides a structured educational context in which practitioners can design movement experiences that may shape how learners come to value, prioritise, adapt, and return to movement across time. The argument is therefore not simply that physical education should increase physical activity levels, but that it may cultivate habits that support physical literacy, lifelong movement, and societal wellbeing. The goal is not to claim that Covey offers a theory of physical literacy, nor to suggest that physical education can directly solve global inequities. Rather, the intention is to explore whether Covey's accessible language of habits can help articulate the patterns of thinking, feeling, relating and acting that support lifelong movement and, in turn, contribute to broader educational and social capital.

Covey's work has been influential in leadership and personal development because it offers a coherent account of how enduring habits underpin long-term effectiveness. His Seven Habits are presented as character-based principles that can be cultivated intentionally. Central to the framework is the distinction between the Circle of Concern and the Circle of Influence. While individuals cannot control all external conditions, they can expand their impact by acting within the sphere of choice, relationship, and practice available to them. And although his framework was not developed for physical education or physical activity, its emphasis on agency, purpose, relationality, and renewal resonates with contemporary physical literacy scholarship. Read heuristically, the habits offer an accessible vocabulary for patterns of thinking, feeling, relating and acting already implicit in physical literacy discourse: taking ownership of movement, orienting engagement toward long-term wellbeing, prioritising what matters, acting inclusively, listening carefully, working with others, and attending to ongoing renewal. Used in this way, Covey's framework does not replace physical literacy theory; rather, it helps render some of its pedagogical and behavioural implications more explicit.

**Figure 1:**
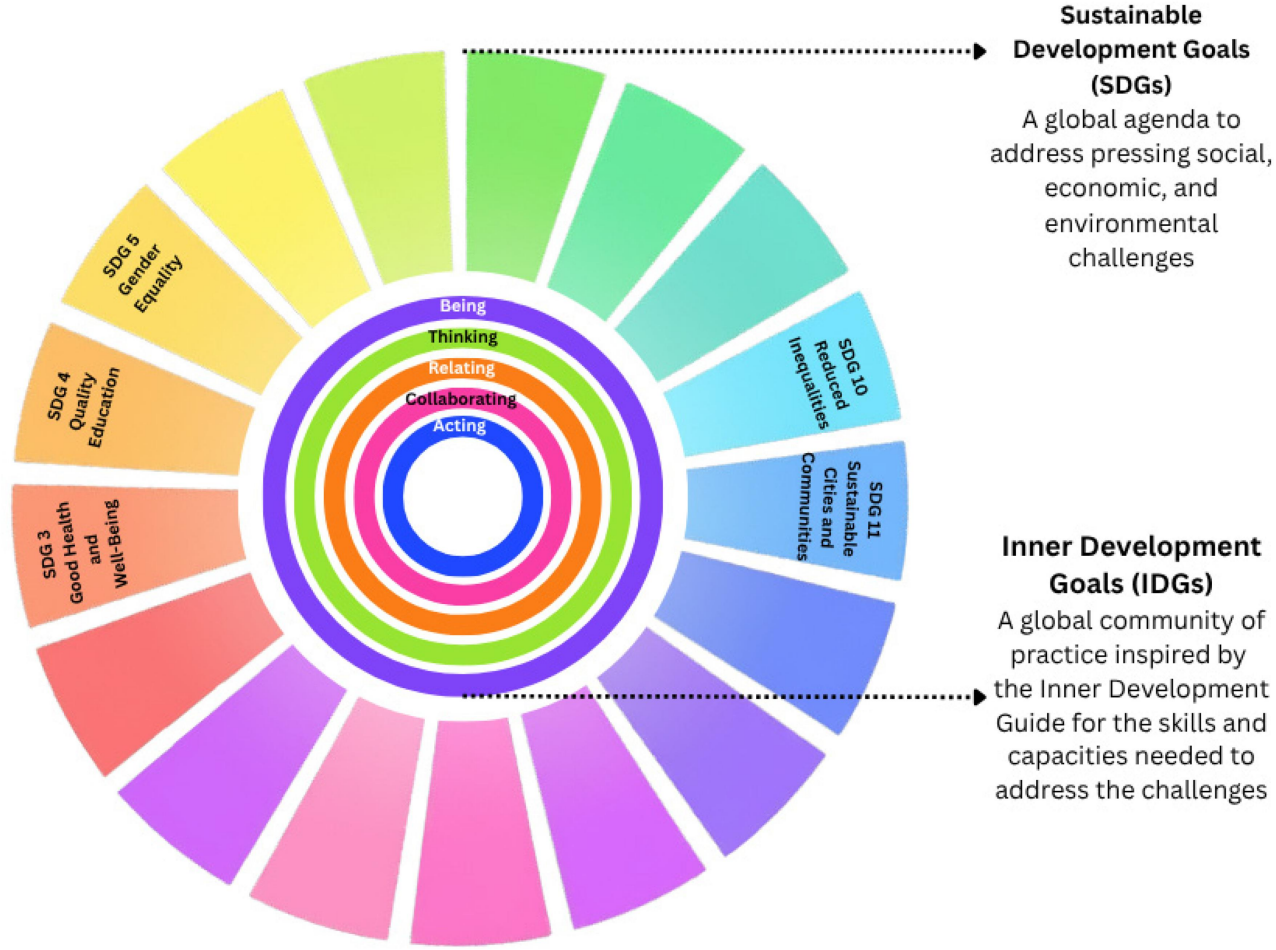
Relationships between Sustainable Development Goals (SDGs) and Inner Development Goals (IDGs). Note. Adapted from Inner Development Goals (20) *Who is IDG?*, https://innerdevelopmentgoals.org/about/resources/.

Situating physical literacy within a broader landscape of human development also requires attention to the inner capacities that support action in complex social worlds. The IDGs articulate five interrelated clusters—Being, Thinking, Relating, Collaborating, and Acting—and present them as capacities needed for constructive responses to contemporary global challenges (19). The IDGs highlight that meaningful sustainable societal change starts with inner growth. It provides a shared framework linking personal development to wider societal transformation. By building 25 skills across five dimensions, individuals and organisations can help drive a collective shift toward more sustainable, regenerative systems and support the achievement of the Sustainable Development Goals (See Figure 1).

Many of these capacities overlap with dimensions already central to physical literacy, including self-awareness, confidence, empathy, resilience, ethical engagement, and relational participation (7, 21). The SDGs, in turn, provide a shared global agenda for health, education, equity, and sustainability (4). Movement pedagogies cannot, in isolation, deliver these broad and SDG outcomes (5, 22). They can, however, create local conditions in which learners practise the kinds of embodied, relational, and reflective practices that matter for health and participation (23), including inclusive interaction (22, 24), self-regulation, cooperation, and care for self and others (25, 26). On this reading, physical education is not a direct mechanism for achieving the SDGs, but a modest and meaningful site of contribution.

This multi-level perspective suggests a useful conceptual bridge. Physical literacy informed pedagogy, understood through a habit-informed lens, can be seen as a way of expanding learners' Circle of Influence. By developing recurring patterns of thinking, feeling, relating and acting such as proactivity, purposeful planning, inclusive engagement, collaboration, and holistic self-care in and through movement, educators may strengthen learners' capacity to shape their own movement lives and to participate more constructively in shared social worlds. The relevance of the IDGs lies in highlighting the inner capacities involved; the relevance of the SDGs lies in clarifying why such capacities matter beyond the individual, to society as a whole.

Against this backdrop, a conceptual gap becomes visible. Physical literacy scholarship has increasingly emphasised holism, relationality, and the lifecourse (7, 10, 27). Yet it has not systematically theorised the role of recurring patterns of thinking, feeling, relating and acting in stabilising and renewing a relationship with movement across time, nor has it fully explored how habit language might help connect physical literacy with wider conversations about inner development and social contribution. Existing work on behaviour change, motivation, and social ecology remains vital, but does not on its own capture the patterned, sedimented quality of how people come to live movement as part of everyday life.

The paper therefore aims to: (a) show how habit can illuminate the behavioural foundations of physical literacy without reducing the concept to automaticity; (b) use Covey's Seven Habits as a heuristic for articulating recurring patterns of thinking, feeling, relating and acting and their pedagogical significance; and (c) position this discussion alongside the IDGs and SDGs to illustrate how physical education may contribute, indirectly and incrementally, to broader agendas of health, wellbeing, equity, and participation. The conceptual model advanced here proposes that movement pedagogy can cultivate identifiable means of thinking, feeling, relating and acting; and that these behaviours can help sustain physical literacy over time. It also suggests that an expanded Circle of Influence can connect personal movement engagement with a wider social contribution.

It should be noted, that the argument that follows is conceptual rather than empirical. It is offered to open further theoretical and practical conversation about how physical literacy is formed, lived, interrupted, renewed, and sustained across the lifecourse. In that sense, the paper invites educators, coaches, researchers, and policy actors to attend not only to what physical literacy includes, but to how it becomes habituated in everyday life.

## Physical literacy, habits, and lifecourse movement

Physical literacy is widely understood as a holistic construct that integrates physical, cognitive, affective, and social dimensions of human development (7, 28). It is also widely described as lifelong, non-linear, and context-sensitive, with periods of growth, regression, reorientation, and renewed engagement across the lifecourse (11, 29). For that reason, physical literacy is often better imagined as a lived journey than a stable status, and one that can be interpreted through biographical mapping and other approaches attentive to transition, identity, and changing circumstance (10, 30).

Habit theory suggests that repeated behaviours enacted in recurring contexts become increasingly stable and, in some circumstances, relatively automatic (13, 14). Gardner (31) emphasises that habits are cue–behaviour associations that can support behavioural maintenance, while identity-based perspectives remind us that people are more likely to enact behaviours that align with who they understand themselves to be (17, 32). Taken together, these perspectives suggest that sustained movement participation is underpinned not by skill or knowledge alone, but by recurring patterns of thinking, feeling, relating, and acting. For example, seeing oneself as someone who moves, protecting time for activity, seeking meaningful challenge, or engaging others in inclusive ways. Recent work linking habit strength and physical activity supports the importance of this line of thinking for maintenance over time (33).

From this perspective, physical literacy can be considered through recurring patterns of thinking, feeling, relating and acting such as agency, reflection, inclusivity, collaboration, and renewal. That claim should be handled carefully. The argument is not that physical literacy is equivalent to habit, nor that embodied, relational, and existential dimensions can be reduced to behavioural repetition. Rather, a habit lens helps illuminate how values, meanings, competencies, and identities become sedimented in everyday practice and come to orient future engagement with movement (8, 34).

A further clarification is important. In this paper, habit refers to recurring patterns of thought and action that become more stable through repetition, context, and meaning (12, 14). In physical education and movement contexts, this may include repeatedly protecting time for movement, seeking meaningful challenge, adapting when participation is interrupted, or returning to movement after periods of disengagement. Rather than using habit to suggest mindless routine, this paper uses a habit-informed lens to examine how cognitive, emotional, social, and behavioural patterns can support the continuity of physical literacy across time. Covey's Seven Habits are therefore not treated as a substitute theory of physical literacy, but as a practical vocabulary for naming recurring patterns that are already relevant to physical education pedagogy, including agency, purpose, prioritisation, inclusion, empathy, collaboration, and renewal. Conceptualizing physical literacy as a relationship with movement, therefore, requires attention to the repeated ways people come to notice, interpret, and respond to movement opportunities in everyday life. Choosing to be active, valuing inclusive participation, seeking meaningful challenge, adapting to interruption, and reflecting on one's development are not single acts. Instead, they are recurrent orientations that can become more or less established over time. In that sense, the familiar principles often associated with physical literacy (i.e., holism, inclusion, empowerment, personalisation, variety, and reflection), can be read both as pedagogical design principles and as recurring learner patterns that may develop through repeated experience. This framing prepares the ground for using Covey's Seven Habits as one structured way of describing and cultivating those dispositions.

## Theoretical anchors: Covey's habits, IDGs, and SDGs

Covey's (18) work on habits provides a useful bridge between general habit theory and the patterns of thinking, feeling, relating and acting discussed in physical literacy scholarship. In his framework, habits are not merely routines; they are organised ways of thinking and acting that can be cultivated intentionally. This emphasis on intentional formation sits productively alongside psychological accounts of habit and identity that explain how repeated behaviour, self-perception, and context interact over time (16, 17). In movement pedagogy, Covey's framework is best treated as a heuristic scaffold rather than a comprehensive explanatory model. Its value lies in offering a recognisable and pedagogically usable language for patterns of thinking, feeling, relating, and acting that matter for physical literacy. As Martin (35) suggested in relation to physical education, such a framework can highlight proactive, relational, and reflective ways of teaching and learning. It can also help connect everyday pedagogical choices with longer-term aims of participation, wellbeing, and human flourishing.

This use of Covey also warrants brief epistemological clarification. Covey's framework does not emerge from physical literacy scholarship, nor is it culturally neutral; rather, it derives from a popular self-development tradition shaped by particular normative and organisational assumptions. It is therefore not adopted here as foundational theory or as a universal account of human development. Instead, the framework is used in a pragmatist educational sense as a heuristic device whose value lies in whether it helps render complex ideas more intelligible and pedagogically actionable for practitioners (36). This is especially important in relation to physical literacy, where conceptual depth must be preserved without allowing the concept to become so diffuse or abstract that its educational implications are difficult to communicate or enact (34). The justification for drawing on Covey, then, is not that his model is exhaustive or context-free, but that its accessible language offers a translational vocabulary for naming patterns of thinking, feeling, relating and acting already implicit within physical literacy scholarship, while remaining clearly subordinate to the richer philosophical foundations on which physical literacy rests.

The Inner Development Goals foreground the internal capacities people need in order to respond constructively to complex global challenges. Specifically, the framework emphasises self-awareness, presence, critical reflection, empathy, communication, courage, and purposeful engagement (19). In educational terms, the significance of the IDGs lies in their shift from a narrow focus on content acquisition toward a concern with the kinds of persons educational processes help to cultivate (37).

The IDGs and SDGs operate at different but complementary levels. The SDGs describe global priorities and desired societal outcomes, including health, education, equity, and sustainable communities. The IDGs focus on the inner capacities that may help individuals and groups act constructively in relation to those priorities. In this paper, the IDGs are therefore not treated as another outcome framework for physical literacy. Rather, they help name the kinds of inner capacities, such as self-awareness, perspective-taking, collaboration, courage, and purposeful action, that can be practised through meaningful movement experiences in physical education. The SDGs, in turn, clarify why those capacities matter beyond the individual: they connect physical education and physical literacy to broader concerns about public health, inclusive education, social equity, and community wellbeing.

Physical literacy shares this developmental orientation. Its affective and social dimensions already point toward confidence, self-regulation, empathy, resilience, ethical engagement, and relational participation in and through movement (7, 21, 28). Physical education can therefore be understood as a distinctly embodied site for nurturing IDG-aligned capacities: listening to one's body and emotions relates to Being; problem-solving in games and creative tasks relates to Thinking; negotiating roles, rules, and space with others relates to Relating and Collaborating; and taking responsibility for participation and wellbeing relates to Acting. This connection does not make PL synonymous with the IDGs, but it does suggest a meaningful area of overlap.

The United Nations Sustainable Development Goals provide a shared framework for thinking about health, education, equity, and sustainable futures (4). Several goals are especially relevant to physical education, physical literacy, and lifelong movement: SDG 3 Good Health and Well-Being; SDG 4, Quality Education; SDG 5, Gender Equality; SDG 10, Reduced Inequalities; and SDG 11, Sustainable Cities and Communities. These goals do not make physical education responsible for solving global challenges. Rather, they reinforce why inclusive movement opportunities, meaningful participation, and lifelong engagement matter within wider social and policy agendas.

The contribution of physical literacy to these agendas is necessarily indirect. Physical education does not produce social justice or sustainability by itself. What it can do is cultivate recurring participatory patterns and relational capacities (e.g., regular engagement, respect for difference, cooperation, self-regulation, and shared responsibility) that support healthier and more inclusive communities over time. In that sense, physical literacy offers a plausible pathway through which local and individual movement practices can contribute, modestly but meaningfully, to wider societal goals.

### Integrative framing

Education provides one of the principal settings in which habits, identities, and capacities are repeatedly formed and rehearsed (38). Within this broader educational landscape, physical literacy can be positioned as a bridge concept linking individual movement throughout life with broader questions of health, participation, and social wellbeing (11). An integrative framing therefore becomes possible: Covey's habits offer a vocabulary for patterns of thinking, feeling, relating and acting; the IDGs help name the inner capacities these patterns require and cultivate; and the SDGs clarify the wider social relevance of such capacities.

This relationship should not be imagined as linear or guaranteed. Not every pedagogical effort will produce durable habits, and not every individual disposition scales neatly to collective change. Even so, the model is useful because it shows how micro-level pedagogical practices (e.g., goal-setting, reflection, cooperative problem-solving, attention to learner voice, and purposeful opportunities for agency) may support the development of patterns and capacities that matter for both lifelong movement and wider educational aspirations. From this perspective, physical literacy is not simply an outcome of education but one mechanism through which embodied learning can contribute to broader forms of human and social development. More specifically, the relevance to sustainable development lies not in claiming that movement pedagogy directly delivers macro-level change, but in arguing that repeated embodied practices can cultivate dispositions that travel beyond the lesson itself. When learners come to repeatedly practice agency, cooperation, inclusion, empathy, and renewal in movement settings, they are not only sustaining their own relationship with movement; they are also rehearsing forms of participation that matter for healthier, more equitable, and more socially connected communities.

## Mapping Covey’s habits onto physical literacy pedagogy

Before considering each habit in turn, it is important to reiterate that Covey's framework is being used here as a heuristic scaffold rather than as a definitive model of physical literacy. The value of the Seven Habits does not lie in their status as an empirically derived theory of movement behaviour, nor is there any claim that they map neatly onto established domains of physical literacy. Rather, their usefulness is conceptual and pedagogical: they provide a recognisable language through which to examine recurring patterns of thinking, feeling, relating and acting that may help explain how engagement in movement is initiated, sustained, and renewed across time and context. Read in this way, the “habits” offer a pragmatic vocabulary for bringing into sharper focus patterns and capacities already implicit within physical literacy scholarship, including agency, purpose, prioritisation, inclusion, empathy, collaboration, and holistic renewal. The purpose of the mapping that follows, then, is not to reduce physical literacy to a set of prescriptive traits, but to explore how a habit-informed lens might illuminate the patterned ways of thinking, relating, and acting that support an ongoing relationship with movement across the lifecourse. As such, Covey's “habits” are treated less as automatic routines than as pedagogically recognisable names for recurring patterns that may become stabilised through repeated practice.

The following mapping retains the specific connection between each of Covey's Seven Habits, selected IDG capacities, and selected SDGs. These connections are not intended as causal claims that a single physical education practice directly produces sustainable development outcomes. Rather, they are offered as illustrative links showing how particular pedagogical patterns in physical education may support inner capacities and broader social aims. In this sense, the habits provide a practical vocabulary for considering how physical education can contribute modestly and indirectly to lifelong movement, wellbeing, inclusion, and social participation.

### Habit 1: be proactive—agency and self-directed movement

Within a habit-informed framing, be proactive, resonates strongly with physical literacy's emphasis on agency and self-directed movement. It points to a recurring pattern of noticing opportunities, taking responsibility, and acting within the sphere of what can be influenced. This aligns with evidence that autonomous, self-endorsed behaviour is central to sustained physical activity engagement (39, 40). When learners repeatedly initiate movement for themselves (i.e., choosing to participate, adapt tasks, persist through challenge, or re-engage after interruption), such actions can become more stable features of how they approach movement in everyday life (14).

Pedagogically, be proactive can be cultivated through choice-rich tasks, opportunities for self-directed practice, and structures for goal-setting, reflection, and self-monitoring. Approaches such as Cooperative Learning (41) and Sport Education (42) are useful here because they ask learners to assume meaningful responsibility for participation rather than simply following instruction. Framed through the IDGs, this habit aligns with self-awareness and agency within the Being and Acting clusters. Its relevance to SDG 3 is indirect but important: physical education experiences that repeatedly invite learners to take initiative, adapt, and re-engage may support health-oriented patterns that extend beyond the lesson itself. In this way, proactivity matters not only because it supports participation in movement, but because it helps learners recognise themselves as capable of acting within their own Circle of Influence.

### Habit 2: begin with the end in mind—lifelong movement orientation

Begin with the end in mind, can be read, in a physical literacy context, as cultivating a recurring pattern of engaging in movement with a long-term and value-informed horizon. Goal-setting and future-oriented thinking matter most when they are linked to how individuals understand themselves and their possible futures as movers (17, 43). Applied to physical literacy, this means orienting participation not toward short-term performance alone, but toward an ongoing relationship with movement that supports enjoyment, competence, connection, and wellbeing across the lifecourse.

Pedagogically, this habit invites educators to help learners develop movement identities, personally meaningful aims, and opportunities to interpret current participation as part of a larger movement biography. Strategies such as self-referenced assessment, reflective journaling, and learner-centred planning can support that work because they connect present experience to future possibility (10). Within the IDGs, this habit aligns with purpose, long-term orientation, and intentional action. Its relevance to SDG 3 and SDG 4 lies in the way physical education can help learners connect present participation with longer-term wellbeing, learning, and life possibilities. This does not mean that physical education guarantees lifelong movement, but that it can provide structured opportunities for learners to imagine, plan for, and value movement as part of a meaningful life.

### Habit 3: put first things first—prioritising meaningful movement

Put first things first, addresses the challenge of sustaining movement amid competing demands. In a physical literacy context, it involves the gradual development of self-regulatory patterns of thinking, feeling, relating and acting through which individuals learn to protect time, attention, and energy for meaningful movement, even when other pressures intervene (31, 44). Physical literacy, framed this way, is not only about knowing how to move, but about repeatedly giving movement a valued place in everyday life.

Pedagogically, this habit shifts attention from maximising activity time or performance outputs to designing movement experiences that feel worthwhile enough to be prioritised. The physical literacy Consensus Statement for England emphasises holism, inclusion, empowerment, and person-centred practice, all of which support learners in seeing movement as relevant to their own lives and wellbeing (45). Lessons that build in choice, variety, and reflection can help learners notice why movement matters and why planning and decision-making are needed to keep physical activity present in daily routines. Viewed through the IDGs, this habit cultivates self-regulation, discipline, and purposeful action within the Being and Acting clusters. Its relevance to SDG 3 lies in helping learners practise ways of giving movement a valued place in everyday life. Where physical education is inclusive and responsive, this habit may also support the broader educational aims reflected in SDG 4 by helping learners develop the reflective and practical capacities needed to sustain meaningful engagement across time.

### Habit 4: think win–win—inclusive movement environments

Think win-win, foregrounds a habit of seeking mutually beneficial outcomes. In physical literacy-focused pedagogy, this maps onto the creation of inclusive movement environments in which success is not defined as the defeat of others but as meaningful participation and development for all learners (23, 46). Such a pattern aligns closely with physical literacy's emphasis on inclusion, empowerment, and person-centred practice.

Pedagogically, think win-win can be cultivated through cooperative structures, adaptable tasks, and success criteria that recognise collaboration, effort, care, and personal progress alongside achievement. When learners repeatedly encounter norms of fairness, mutual encouragement, and shared problem-solving, those norms can become established habits that shape how they relate to others in movement settings. In the context of the IDGs, this habit aligns with empathy, inclusive mindset, trust, and collaboration. Its relevance to SDG 5 and SDG 10 lies in the way physical education can challenge exclusionary norms and create conditions in which different learners can participate meaningfully. The connection is not that a single lesson produces equity, but that repeated experiences of shared success, mutual recognition, and adaptive participation may help learners practise more inclusive ways of moving with others.

### Habit 5: seek first to understand—learner-centred pedagogy

Seek first to understand, aligns closely with learner-centred physical literacy pedagogy because it begins with attentive listening to learners' experiences rather than imposing a uniform starting point. In practice, this means taking seriously children's movement biographies, motivations, fears, exclusions, and aspirations, and using that understanding to shape meaningful and equitable participation opportunities (23, 38).

Pedagogically, the habit is expressed through student voice, dialogic teaching, co-construction of tasks, and responsive adaptation. This matters for physical literacy because the development of confidence, motivation, and participation is closely tied to whether learners feel heard, recognised, and supported. Through the IDGs, this habit aligns with perspective-taking, humility, empathy, and compassion. Its relevance to SDG 4 and SDG 10 lies in the way learner-centred physical education can widen access to meaningful participation by beginning with learners' experiences rather than assuming a universal starting point. Listening, adaptation, and responsiveness are therefore not only pedagogical strategies; they are practices through which inclusion is actively produced.

### Habit 6: synergise—social learning and community movement cultures

Synergise, captures a recurring pattern of creative cooperation and collaboration that sits at the heart of the social dimensions of physical literacy. Social learning perspectives remind us that development unfolds through shared activity, dialogue, modelling, and joint problem-solving rather than in isolation (47). In movement contexts, this means that identity formation is often relational. In other words, learners come to see themselves as movers partly through the ways they participate with, are recognised by, and contribute to others.

Pedagogically, synergise can be cultivated through group problem-solving tasks, peer mentoring, co-designed games, and community-oriented movement projects. Such practices embody the principle that physical literacy is expressed not only in moving well alone, but in moving well with others. With respect to the IDGs, this habit aligns with collaboration, communication, co-creation, and collective problem-solving. Its relevance to SDG 3 and SDG 11 lies in the way physical education can help learners experience movement as a shared social practice rather than an individualised accomplishment. By cultivating cooperation, mutual contribution, and collective responsibility, physical education may support forms of relational wellbeing that matter for healthier and more connected communities.

### Habit 7: sharpen the saw—holistic renewal and lifelong wellbeing

Sharpen the saw, aligns closely with the holistic orientation of physical literacy because it foregrounds ongoing renewal across physical, emotional, social, and cognitive dimensions of life (7, 10, 48). Read in this way, movement is not only exercise or performance; it is also a practice of caring for the whole person through enjoyment, stress management, connection, rest, recovery, and meaning (5, 28, 49).

Pedagogically, this habit points toward movement programmes that integrate reflection, recovery, and wellbeing literacy alongside participation. Learners can be invited to notice how different forms of activity affect mood, energy, connection, and self-understanding, and to adjust their participation accordingly. Against the backdrop of the IDGs, this habit aligns with self-awareness, balance, resilience, and sustainable action. Its relevance to SDG 3 is especially clear, as physical education can help learners understand movement in relation to recovery, wellbeing, stress management, connection, and care for self and others. Its broader relevance to SDG 4 lies in treating physical education as a site of holistic learning rather than simply physical performance. Read in this way, renewal is not a private luxury but part of learning how to sustain movement, wellbeing, and participation across the lifecourse.

Across the seven habits, the links to the IDGs and SDGs should be understood as cumulative and pedagogical rather than linear or deterministic. Each habit highlights a different pattern that physical education may help cultivate: agency, purpose, prioritisation, inclusion, empathy, collaboration, and renewal. Together, these patterns suggest how physical education can contribute to physical literacy in ways that extend beyond immediate participation. The contribution remains modest, but meaningful: physical education can provide repeated opportunities for learners to practise ways of moving, relating, choosing, adapting, and reflecting that may support lifelong movement and broader forms of individual and collective wellbeing.

## Conceptual model: a habit-informed reframing of physical literacy

In the conceptual model proposed here, physical literacy is understood as an ongoing relationship with movement shaped through recurring movement-related patterns that develop and become progressively stabilised through repeated experience, reflection, social interaction, and participation across time. Movement trajectories are shaped by biography and context, but habits can provide a degree of continuity within that variability by stabilising how people think about, value, prioritise, and return to movement (14, 49). Identity processes further reinforce this continuity, because people are more likely to sustain practices that fit with who they take themselves to be (17, 32). In this sense, physical literacy is not treated as a fixed state or endpoint, but as an ongoing and context-sensitive relationship with movement that is expressed through recurring patterns such as agency, purpose, prioritisation, inclusion, empathy, collaboration, and renewal.

**Figure 2:**
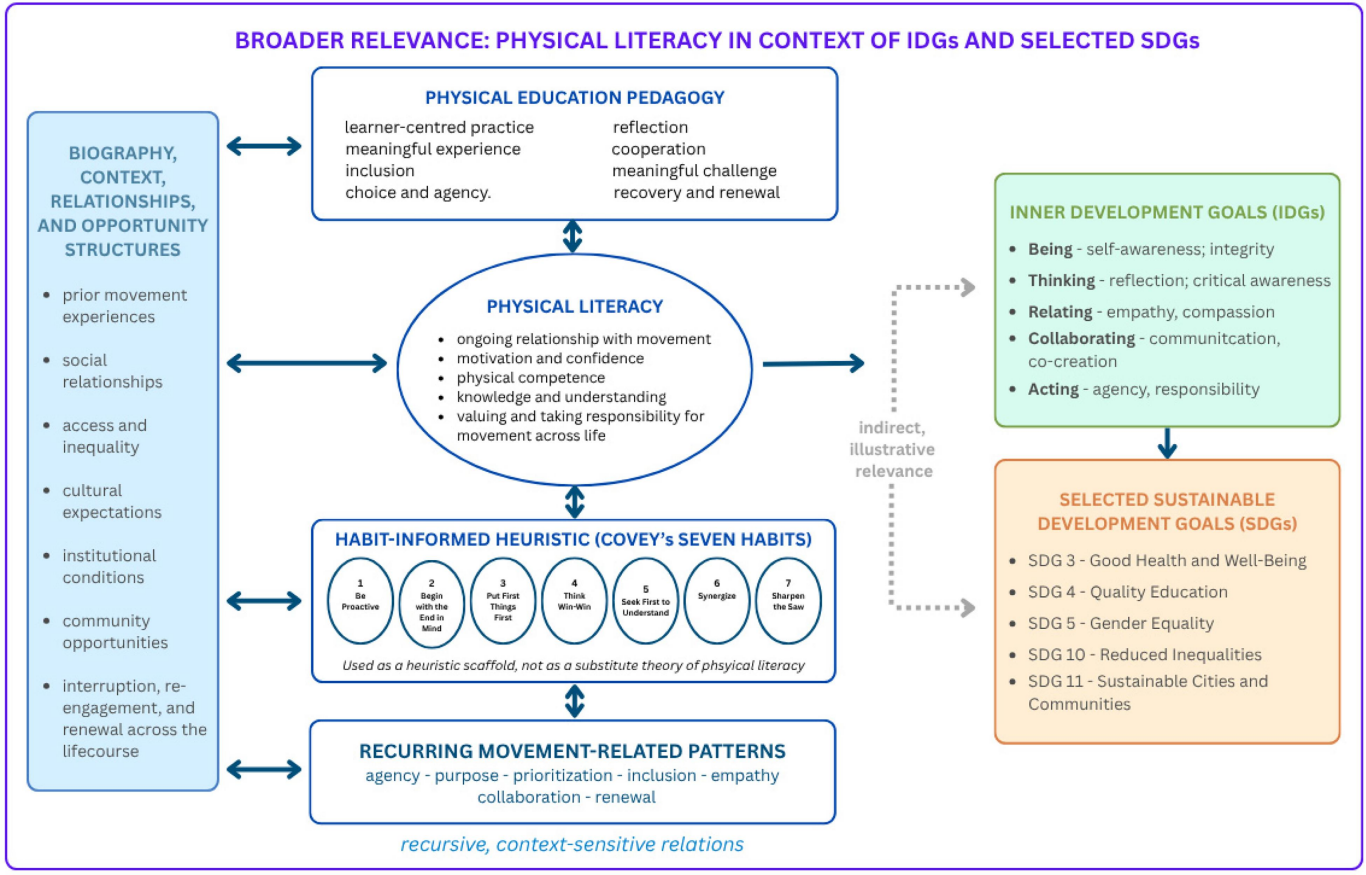
Physical literacy in context: a habit-informed model of lifelong movement and societal relevance. *Note.* Physical literacy is presented as an ongoing, context-sensitive relationship with movement. Covey's seven habits are used as a heuristic vocabulary for naming pedagogically relevant patterns, not as a substitute theory of physical literacy. The IDGs and selected SDGs are shown as the broader context of relevance rather than as linear endpoints.

Figure 2 visualises this argument as a layered and recursive model organised across four interrelated elements: physical education pedagogy, physical literacy, heuristic articulation, and recurring movement-related patterns. These elements are situated within two broader contextual frames. The first is the lifecourse context of biography, relationships, access, culture, institutions, community opportunities, and experiences of interruption, renewal, and re-engagement. The second is the wider ethical and societal context represented by the Inner Development Goals and selected Sustainable Development Goals. In this way, the model does not position the IDGs or SDGs as outcomes at the end of a linear pathway. Rather, they frame the broader relevance of physical literacy by clarifying the kinds of inner capacities and societal priorities to which physical education may modestly and indirectly contribute.

At the level of pedagogical formation, the model places physical education pedagogy in direct recursive relation with physical literacy. This reflects the argument that physical literacy is not merely an outcome of pedagogy, but is continually formed and re-formed through learner-centred, cooperative, reflective, inclusive, and meaningful movement experiences designed by teachers, coaches, and practitioners (22, 26). The bidirectional connection between physical education pedagogy and physical literacy signals that pedagogical practice shapes how learners come to experience, value, and take responsibility for movement, while developing physical literacy may also feed back into renewed participation, greater agency, and more self-directed engagement in movement settings. In this sense, physical education can expand learners' Circle of Influence by helping them recognise where they can act, adapt, participate, and contribute within personal, relational, and community contexts.

Heuristic articulation represents the interpretive level at which Covey's Seven Habits are used as a pedagogically accessible vocabulary for naming movement-relevant patterns. The habits are not treated as rigid behavioural routines or as a substitute theory of physical literacy. Rather, they are used as heuristic language for articulating recurring patterns such as agency, purpose, prioritisation, inclusion, empathy, collaboration, and renewal. The vertical connection between physical literacy, Covey's habit-informed heuristic, and recurring movement-related patterns highlights the paper's central claim that repeated movement experiences may, over time, help stabilise how learners think, feel, relate, and act in relation to movement. These patterns, in turn, shape how learners encounter future movement opportunities, interpret challenges, respond to interruption, and re-engage across the lifecourse.

The broader relevance of the model is represented through the IDGs and selected SDGs. The IDGs identify inner capacities that overlap with the kinds of capacities physical education may help learners practise through movement, such as self-awareness, reflection, empathy, communication, agency, and responsibility. The selected SDGs, identify broader societal priorities related to health and wellbeing, quality education, gender equity, reduced inequalities, and sustainable communities. The model deliberately presents these frameworks as a context of relevance rather than as direct outcomes. It does not suggest that physical education or physical literacy can, by themselves, deliver macro-level social change. Instead, it suggests that physical education may cultivate recurring movement-related patterns and capacities that are relevant to healthier, more equitable, and more socially connected communities over time.

Importantly, the model should not be read as a simple sequence in which one stage mechanically produces the next. Rather, it is best understood as recursive and context-sensitive. The arrows indicate broad developmental relations across the model, but they also show feedback, return, and re-shaping. The contextual element of biography, context, relationships, and opportunity structures signals that movement is always lived within social, relational, institutional, and material conditions. These include prior movement experiences, social relationships, access and inequality, cultural expectations, institutional conditions, community opportunities, and experiences of interruption, renewal, and re-engagement across the lifecourse. The model therefore links physical education pedagogy, physical literacy, heuristic articulation, recurring movement-related patterns, and wider societal relevance while preserving the non-linear, situated, and evolving character of lifelong movement engagement.

### Implications

This reframing has implications for curriculum design, pedagogy, teacher education, assessment, and future research. For physical education practice, the central implication is that physical literacy should be supported not only through the development of competence, knowledge, or motivation, but also through repeated movement experiences that help learners build durable patterns of agency, reflection, inclusion, collaboration, and renewal. Physical education should therefore be designed to make meaningful participation possible across different abilities, identities, contexts, and life stages. In curriculum and programme design, this means treating continuity, adaptation, and re-engagement as explicit aims rather than incidental outcomes of participation. Assessment may also need to move beyond narrow skill checklists toward approaches that make biography, agency, reflection, and lived continuity more visible (10, 34, 50).

For teachers and practitioners, a habit-informed perspective offers a reflective vocabulary for examining whether physical education practices repeatedly invite the patterns associated with lifelong movement. Choice-rich tasks, cooperative learning, self-referenced goals, student voice, and opportunities for reflection can help learners connect present movement experiences to longer-term identities and possibilities as movers. The practical contribution of this reframing is not that practitioners should explicitly teach Covey's Seven Habits, but that the habits can help them consider whether their pedagogical aims and practices support agency, purpose, prioritisation, empathy, collaboration, and renewal.

For research, the paper points to several priorities. Conceptually, future work should continue to clarify how habit, identity, embodiment, and physical literacy intersect without reducing physical literacy to automatic behaviour or individual responsibility alone (8, 34, 51). Methodologically, longitudinal, qualitative, and mixed-methods designs are needed to examine how movement-related patterns are formed, interrupted, re-established, and sustained across different life stages and settings. This includes research on how learners carry physical education experiences into later movement contexts, and how biography, relationships, access, and opportunity shape the continuity of physical literacy. Empirically, future studies could examine whether patterns such as proactivity, prioritisation, inclusion, collaboration, and renewal help explain maintenance, dropout, and re-engagement in movement in ways that complement existing motivational and social-ecological models (11, 33, 49). Research could also examine how physical literacy-oriented pedagogies align with the inner capacities emphasised in the IDGs and with broader social aims reflected in selected SDGs.

## Conclusion

Physical literacy is best understood as an embodied, evolving, and context-sensitive process that reflects an individual's ongoing relationship with movement across the lifecourse (6, 28). This paper has argued that a habit-informed lens can deepen that understanding by drawing attention to the patterns of thinking, feeling, relating and acting through which movement is noticed, valued, prioritized, enacted, interrupted, and renewed. These patterns are not formed in isolation. They are shaped by biography, meaning a person's accumulated movement experiences across life; by context, including the social, cultural, institutional, and material conditions in which movement occurs; by relationships, including the people who support, constrain, recognise, or discourage participation; and by opportunity structures, including access to safe spaces, inclusive programmes, time, resources, and community supports. Reframed in this way, physical literacy is not a finished achievement or individual trait, but a lived relationship with movement that is continually shaped by personal history and social conditions.

Using Covey's Seven Habits as a heuristic scaffold, and situating that scaffold in relation to the IDGs and SDGs, the paper has proposed a habit-informed reframing of physical literacy that connects physical education, lifelong movement, and broader questions of health, education, inclusion, and community wellbeing. The central claim is deliberately modest: physical education cannot solve inactivity or inequality on their own, but it can help cultivate patterns of thinking, feeling, relating and acting that matter for lifelong movement and collective wellbeing. Those patterns matter because they shape how people care for themselves, include others, share space, cooperate across difference, and sustain meaningful participation within communities. Reframed in this way, physical literacy becomes not only a language for describing movement engagement, but a way of thinking about how physical education might contribute to reflective, relational, and responsible lives.

## Data Availability

The original contributions presented in the study are included in the article, further inquiries can be directed to the corresponding author.
